# Supplementary material for: Altered microRNA Transcriptome in Cultured Human Liver Cells upon Infection with Ebola Virus
Source: Int J Mol Sci. 2021 Apr 6;22(7):3792. doi: 10.3390/ijms22073792 (PMC8038836; doi:10.3390/ijms22073792)
Supplement: Supplementary file 1 [file ijms-22-03792-s001.zip › Supplementary_File/C_ GO_Analysis_Results/16-30nt_go_Mayinga-24h-Huh7_vs_Control-24h-Huh7_up.mature_mirna_targets/CC_FoldEnrichment.pdf]

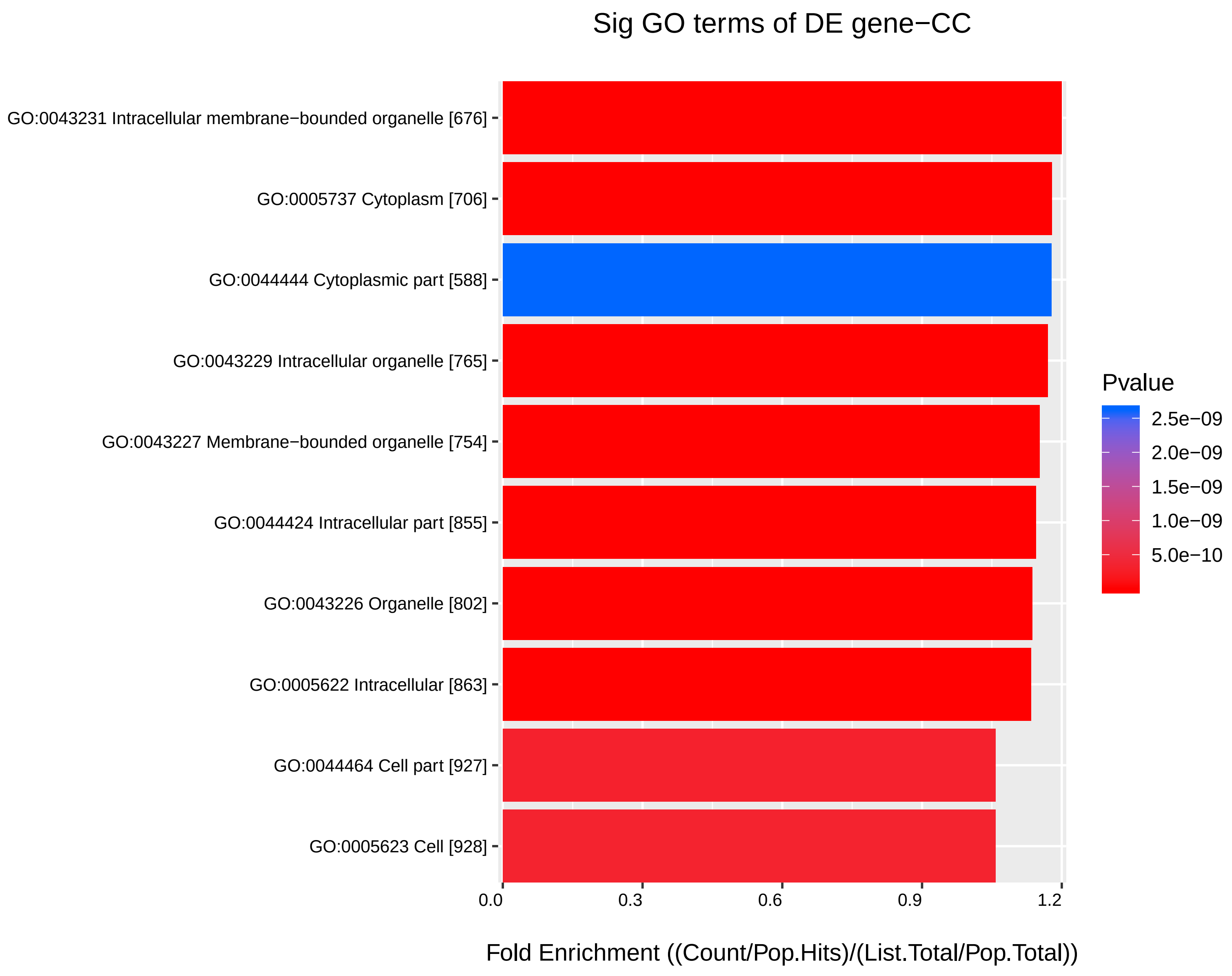
Sig GO terms of DE gene−CC
GO:0043231 Intracellular membrane−bounded organelle [676]
GO:0005737 Cytoplasm [706]
GO:0044444 Cytoplasmic part [588]
GO:0043229 Intracellular organelle [765]
GO:0043227 Membrane−bounded organelle [754]
GO:0044424 Intracellular part [855]
GO:0043226 Organelle [802]
GO:0005622 Intracellular [863]
GO:0044464 Cell part [927]
GO:0005623 Cell [928]
0.0
0.3
0.6
0.9
1.2
Fold Enrichment ((Count/Pop.Hits)/(List.Total/Pop.Total))
Pvalue
2.5e−09
2.0e−09
1.5e−09
1.0e−09
5.0e−10
